# American Dental Convention

**Published:** 1867-01

**Authors:** W. C. Horne


### AMERICAN DENTAL CONVENTION.

The 13th annual meeting of this body will be held in the city of New York, on Tuesday, the 5th of March next. The executive committee have made arrangements which will ensure an interesting and entertaining session.

On the evening of Wednesday, the 6th of March, the commencement of the N. Y. College of Dentistry will be held in Steinway Hall. The address to the graduating class will be delivered by Dr. W. W. Allport, of Chicago; Hon. John T. Hoffman, Mayor of New York, and other eminent speakers will deliver addresses.

A cordial invitation is hereby extended to Dentists throughout the country, to be present and take part in the proceedings of the Convention.

Further particulars will appear in the N. Y. daily papers.

W. C. HORNE, Sec'y.

